# The interactions of fault patterns and stress fields during active faulting in Central North China Block: Insights from numerical simulations

**DOI:** 10.1371/journal.pone.0215893

**Published:** 2019-04-25

**Authors:** Bo Shao, Guiting Hou

**Affiliations:** Key Laboratory of Orogenic Belts and Crustal Evolution, Education Administration, School of Earth and Space Sciences, Peking University, Beijing, China; Chinese Academy of Geological Sciences, CHINA

## Abstract

The interaction of active faults as a factor affecting the mechanisms of large earthquakes has been observed in many places. Most aftershock and clustering earthquake sequences do not recur on the main seismogenic fault but are controlled by fault interactions with adjacent seismic structures. Four groups of conceptual models were generated in this study to determine how the geometry of the seismogenic faults controls the distributions of stress fields and earthquakes. The influences of the fault length ratio, center distance, overlap ratio, echelon distance and fault opening angle were considered in a 2D viscoelastic model. The results indicate that the interaction in the slipping zone is larger when collinear interacting faults are more closely positioned, with one fault lengthening. For noncollinear faults, the interaction is stronger as the inner tips pass each other, which impedes their growth after some degree of overlap. Additionally, fault interaction at the slipping zone becomes stronger as the opening angle approaches 180°. We further generated a 3D viscoelastic model of fault interactions in Central North China Block and applied the finite element method to analyze the relationship between distributions of earthquakes and fault geometry. The calculated results reveal well-matched higher stress and maximum shear strain concentrations in the southern part of the Fen-wei Graben Zone than in other zones in Central North China Block, which can be explained by the longer faults, shorter center distances, shorter overlap lengths and larger opening angles. The stress distributions and fault interactions should be considered in long-term seismic hazard assessment in these zones.

## Introduction

Interactions between active faults have been observed in many places, and stress-change calculations for such interactions can reveal information about the dynamics and evolution of earthquakes, e.g., in California[1–4]. The motivation behind this study is to shed light on the dynamics of seismic initiation and migration by analyzing the interaction between different faults with various geometries and kinematics[4].

Problems involving fault interactions have received much attention through various analytical methods and physical and digital simulations over the past decades. Early studies focused on the stress regime and propagation mechanism of single faults[5–8]. Interactions between double fractures were then investigated in analytical solutions[9]. Only two collinear fractures were considered in these studies[10]. However, analytical solutions are too simple for real geologic conditions and are not suitable for noncollinear fractures.

The calculation of the required quantities in a viscoelastic structure is a sophisticated problem that necessitates advanced numerical methods[11]. Simulation of fault interactions has also been extensively employed in the previous literature. Physical simulation has been used to study the propagation of single-fracture and double-fracture models[12]. Additionally, the interaction between double strike-slip faults has been studied in physical simulation experiments[13]. Limited in terms of materials and processes, physical simulation modeling is often relatively simple but difficult to fit with the observed geology.

The finite element method (FEM) has been exclusively employed in fault interactions[1]. FEM analysis has also been performed under smooth contact conditions in both two and three dimensions. The influences of fault length, center distance, overlap ratio, en echelon distance and other factors have been considered in the research. However, all these solutions are incomplete and define the displacement and stress fields of only limited fault complexes around the cracks. Predecessors mainly considered one or two factors controlling fault interactions instead of coupling all factors and did not consider the interactions between double faults with different orientations.

In this contribution, multiple coupling factors that influence fault interaction are considered with a viscoelastic 2D FEM application. We further analyze the relationship between the distributions of earthquakes and faults in the Central North China Block based on fault interactions.

## Conceptual modeling

### Construction of the conceptual models

In this paper, we utilize four groups of 2D models with the same set of mechanical properties but different fault combinations to determine the roles of fault interactions using the FEM. The models have been developed within the viscoelastic fracture mechanics framework with 2D assumptions. Each time step is set to 100 years, and the entire modeling process consists of 100 steps. The ANSYS 14.0 (University Version 15.0; www.ansys.com) finite element software package is used to calculate the models.

The viscoelastic rheology is controlled by the Maxwell constitutive equation[14]:

$$\dot{\varepsilon }=\;\sigma /\eta \text{eff}+\;\dot{\sigma }/\text{E}$$ (1)

where $\dot{\varepsilon \;}$ is the strain rate, σ is the differential stress, $\dot{\sigma }$ is the stress rate, E is Young’s modulus reflecting the elastic component, and η_eff_ is the effective viscosity. In the above viscoelastic model, Young’s modulus *E* and Poisson’s ratio *ν* can be calculated using the following equations, which essentially define Hooke’s law in three dimensions[15]:

$$V_{p}\;=\;\sqrt{\left(\lambda +2\mu \right)/\rho }$$ (2)

$$V_{s}\;=\;\sqrt{\mu /\rho }$$ (3)

E=μ(3λ+2μ)/(λ+μ) (4)

ν=λ/2(λ+μ) (5)

where *V*_*p*_ represents the P-wave velocity, *V*_*s*_ represents the S-wave velocity, *ρ* represents rock density, *μ* and *λ* are Lamé parameters, *E* is Young’s modulus, and *ν* is Poisson’s ratio.

Most moderately strong earthquakes in North China occur in the gradient belt between different velocity blocks around the middle crust. The average rock mechanical properties of the 2D models could be regarded as those of the middle crust with a density of 2550 kg/m^3^, Young’s modulus of 30 GPa, Poisson’s ratio of 0.25 and viscosity of 1×10^22^ Pa•s[16, 17]. The coefficient of friction of the faults is taken to be 0.4 throughout our calculations, as the selection of the value was proven to have little effect on the spatial pattern of the Coulomb stress change[18].

**Fig 1:**
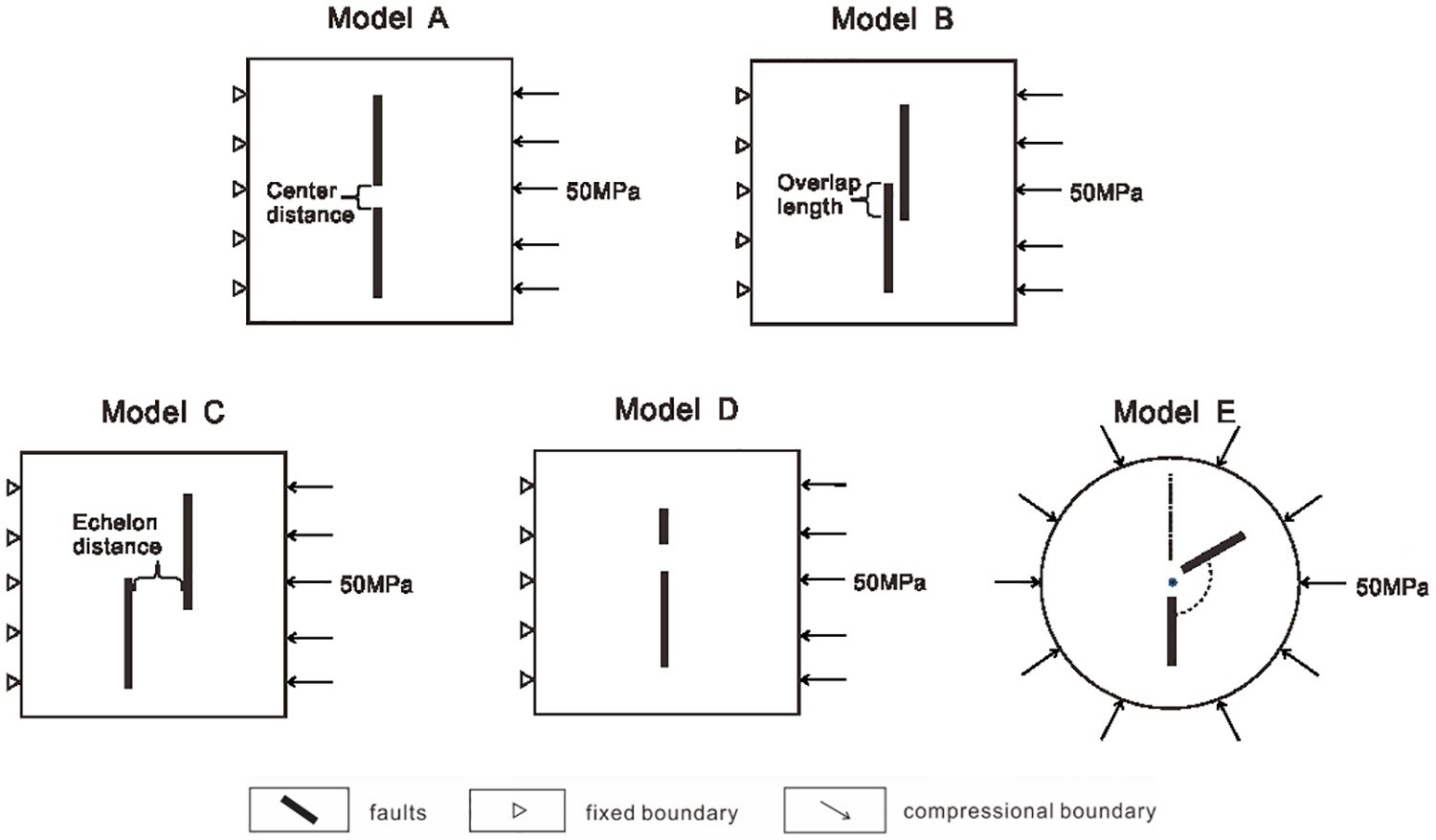
Comparison of model boundary conditions. Model A includes models differing in the center distance. Model B includes models differing in the overlap ratio. Model C includes models differing in the en echelon distance. Model D includes models differing in the length of the upper fault. Model E includes models differing in the fracture opening angle.

The specific geometries considered in this paper are shown in Fig 1. Each group of models considers one factor that is related to fault interaction and control variables to keep the results credible. Model Group A includes five models of collinear faults interacting, which differ in the center distance. The five models of Model Group B are noncollinear parallel faults, which differ in the overlap ratio. Model Group C includes five models of interacting collinear faults, which differ in the length of the upper faults. Model Group D investigates the influence of the fracture opening angle using round models to eliminate errors caused by different distances between the faults and the boundaries.

### Constraints on the conceptual models

In finite element methods, the application of boundary conditions remains one of the key problems[19–23]. In the modeling, four groups of two-dimensional viscoelastic models of fault interaction are employed. In each group, five models are employed with different parameters of faults (Table 1). In our first four groups of conceptual numerical models (Models A, B, C and D), boundary conditions are applied as follows: the up and down boundaries of the model are set free, i.e., all stress components are nil. The left boundary is vertically and horizontally fixed to velocity zero. A 50 MPa compressive deviatoric stress is set on the right boundary. In the last group of conceptual numerical models on the influence of fracture opening angle (Model E), a 50 MPa compressive deviatoric stress is set on the circumference to simulate the extensional or compressional environment, in order to reduce the difference in results caused by the difference in load direction. To verify the stress field under different stress conditions, we also set a 50 MPa tensile deviatoric stress on the right boundary in a group of comparative trials.

**Table 1:** Parameters of faults in the conceptual models.

Model	Center distance (km)	Overlap length (km)	Longer fault length (km)	Shorter fault length (km)	Opening angle (°)
**Model A**	10, 20, 30, 40, 50	0	50	50	180
**Model B**	0	10, 20, 30, 40, 50	50	50	180
**Model C**	20	0	50	10, 20, 30, 40, 50	180
**Model D**	20	0	50	50	60, 90, 120, 150, 180

### Modeling results

#### Center distance

**Fig 2:**
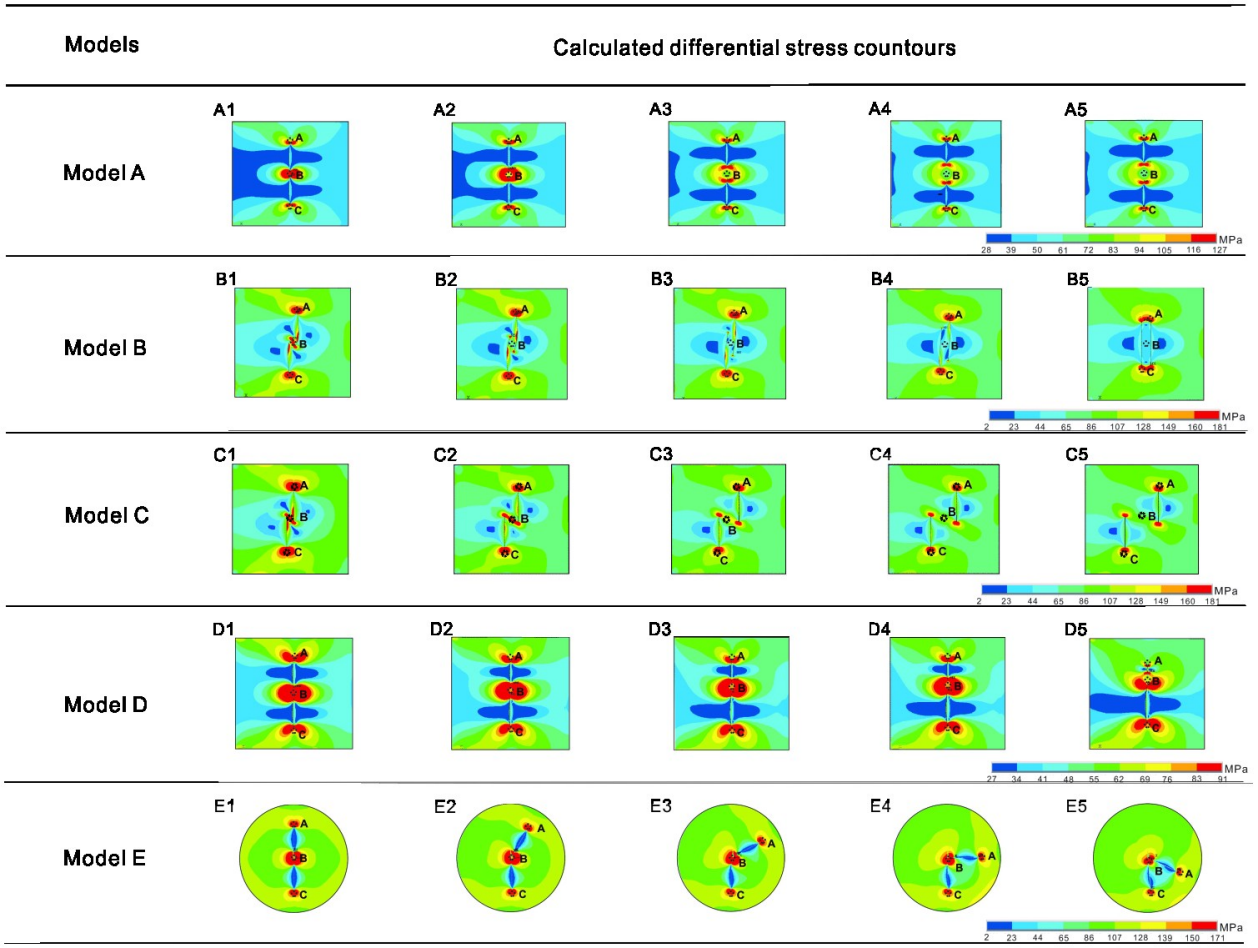
Differential stresses (measured in MPa) calculated by the four groups of models. Black circles denote points A, B and C in each model, where the differential stresses are shown in the curves in Fig 3.

**Fig 3:**
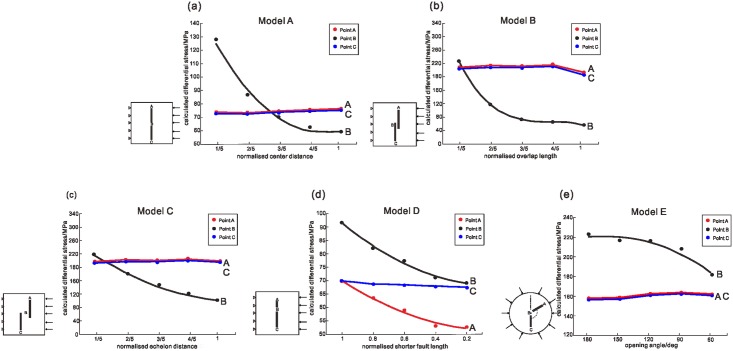
Calculated differential stresses as a function of normalized parameters for the four groups of models. In (a), the variations in the calculated differential stresses at points A, B and C are illustrated as a function of the normalized center distance. In (b), the variations in the calculated differential stresses at points A, B and C are illustrated as a function of the normalized overlap length. In (c), the variations in the calculated differential stresses at points A, B and C are illustrated as a function of normalized en echelon distance. In (d), the variations in the calculated differential stresses at points A, B and C are illustrated as a function of the normalized length of the shorter fault. In (e), the variations in the calculated differential stresses at points A, B and C are illustrated as a function of the opening angles.

The results for the differential stress of two equal-length collinear faults with different center distances are shown in Fig 2-Model A. The center distance is defined as the distance between the inner tips of the double faults. Fig 3(a) illustrates the variations in the calculated differential stress at points A, B and C as a function of normalized center distance for five different separations of the two adjacent faults[24]. For normalization, the center distance is divided by the length L of a single fault of the same length. As shown in Fig 3, in general, the interaction is larger as the interacting faults are more closely positioned. The differential stresses at points A and C represent the influence of the double faults on the far ends. The differential stress at point B represents the influence of the interaction on the zone between the double faults. The curve illustrates that the interaction is significant only when the slipping zones are closer than half of the fault length, which shows good agreement with the experimental results of Bobet and Einstein [12].

#### Overlap length

The results for the differential stress of two equal-length collinear faults with different overlap ratios are shown in Fig 2-Model B. Overlap length is defined as the length of the overlapping portion of the double faults. Fig 3(b) illustrates the variations in the calculated differential stresses at points A, B and C as a function of normalized overlap length for five different separations of the two adjacent faults. For normalization, the overlap length is divided by L of a single fault of the same length[24].

As shown in Fig 3, the differential stresses at points A and C represent the influence of the double faults on the far ends, which remains invariant as the overlap ratio changes. The differential stress at point B represents the influence of the interaction on the zone between the double faults. The results from the curve indicate that fault interaction is stronger as the inner tips pass each other and impedes their growth after some degree of overlap. The calculated differential stress increases sharply as the inner tips approach each other and decreases after the inner tips pass each other to form a larger overlap ratio, which shows good agreement with studies on the San Andreas Fault[1].

#### Echelon distance

The results for the differential stress of two equal-length collinear faults with different echelon distances are shown in Fig 2-Model C. Echelon distance is defined as the orthogonal distance between two echelon faults with some degrees of overlap. Fig 3(c) illustrates the variations in the calculated differential stresses at points A, B and C as a function of normalized echelon distance for five different separations of the two adjacent faults. For normalization, the echelon distance is divided by L of a single fault of the same length[24].

As shown in Fig 3, the differential stresses at points A and C represent the influence of the double faults on the far ends, which remains invariant as the overlap ratio changes. The differential stress at point B represents the influence of the interaction on the zone between the double faults. The results from the curve indicate that fault interaction is stronger as the echelon distance becomes shorter. Only when the echelon distance is approximately one-fifth of the fault length is the calculated differential stress in the overlap area larger than the stress on the far ends, which shows good agreement with studies on the San Andreas Fault[1].

#### Fault lengths

The results for the differential stress of two collinear faults with different fault lengths are shown in Fig 2-Model D. Fig 3(d) illustrates the variations in the calculated differential stresses at points A, B and C as a function of the normalized fault length[24]. For normalization, the upper fault length is divided by L of the invariable lower fault length. As shown in Fig 3, the differential stress at point B represents the influence of the interaction on the zone between the double faults. The results from the curve indicate that fault interaction is stronger as one fault becomes longer. The result emerges that a large preexisting slip zone can reactivate slip and movement along a nearby smaller slip zone. In other words, segmentation of fault slip zones can substantially affect the interpretation of observations, which shows good agreement with experiments[9].

#### Opening angle

The results for the differential stress of two faults with different fault opening angles are shown in Fig 2-Model E. The opening angle is defined as the minimum angle between the two faults. Fig 3(e) illustrates the variations in the calculated differential stresses at points A, B and C as a function of the opening angle[24]. The differential stresses at points A and C represent the influence of the double faults on the far ends, which remains invariant as the opening angle changes. The differential stress at point B represents the influence of the interaction on the zone between the double faults. For normalization, Model Group D uses round models to eliminate errors caused by different distances between the faults and the boundaries. The results from the curve indicate that fault interaction at the slipping zone is stronger as the opening angle is closer to 180°. This result implies that collinear faults are more likely to coalesce than faults with an opening angle.

In short, the results from the curves indicate that fault interactions are stronger with longer fault length, shorter overlap length, shorter center distance and larger opening angle. The coupling of the four factors should be taken into account in the stress distribution and long-term seismic hazard assessment of seismic zones and gaps.

## Fault interaction modeling in Central North China Block

### Seismotectonic setting

The Central North China Block (CNCB) is an old tectonic unit in mainland China, which is also seismically one of the most active intracontinental regions in the world[25–27]. Many previous studies have shown that North China has experienced significant lithospheric thinning since the late Mesozoic[28–31]. The CNCB underwent intensive compression-shear faulting during the earliest Cenozoic[32, 33]. Many NNE- and NE-trending structures passing through the interior of the CNCB developed or were reactivated then[34, 35]. During the early Paleocene–Eocene interval, the NNE- to NE-trending faults were inverted to normal faults, and numerous new normal faults developed. The main seismic belts are oriented in a NNE direction, including the Tangshan–Cixian seismic belt, the Shanxi seismic belt, and the Tan-lu seismic belt. However, the Zhangjiakou-Bohai seismic zone is oriented in a ENE direction.

### Setup of fault interaction model in the CNCB

To reveal how fault interactions controlled stress evolution and earthquakes in the CNCB, a 3D finite element model[36, 37] was applied.

The model was developed within a viscoelastic fracture mechanics framework. Each time step was set to 100 years, and the entire modeling process underwent 100 steps. The model covered a region from 32° to 42°N and 106° to 124°E. The depth of the model was approximately 100 km in accordance with the estimated average lithospheric thickness in the CNCB. The lithosphere was modeled using a layered rheology structure, which included five types of rocks (sediment, upper crust, middle crust, lower crust, and mantle; Table 2). During the calculation process, the selection of the parameters was based on the crustal velocity and viscous structure from previous studies[38] (Table 1).

**Table 2:** Parameters of rock properties in the model[38].

Layer	Thickness (km)	Young’s modulus (GPa)	Poisson’s ratio	Density(10^3^ kg/m^3^)	Viscosity (Pa·s)
**Sediment**	4.2	30	0.25	2.5	10^22^
**Upper crust**	11.1	30	0.25	2.5	10^22^
**Middle crust**	10	30	0.25	2.5	10^21^
**Lower crust**	15	30	0.25	2.7	7.1×10^18^
**Mantle**	60	30	0.25	3.0	2.1×10^19^

**Fig 4:**
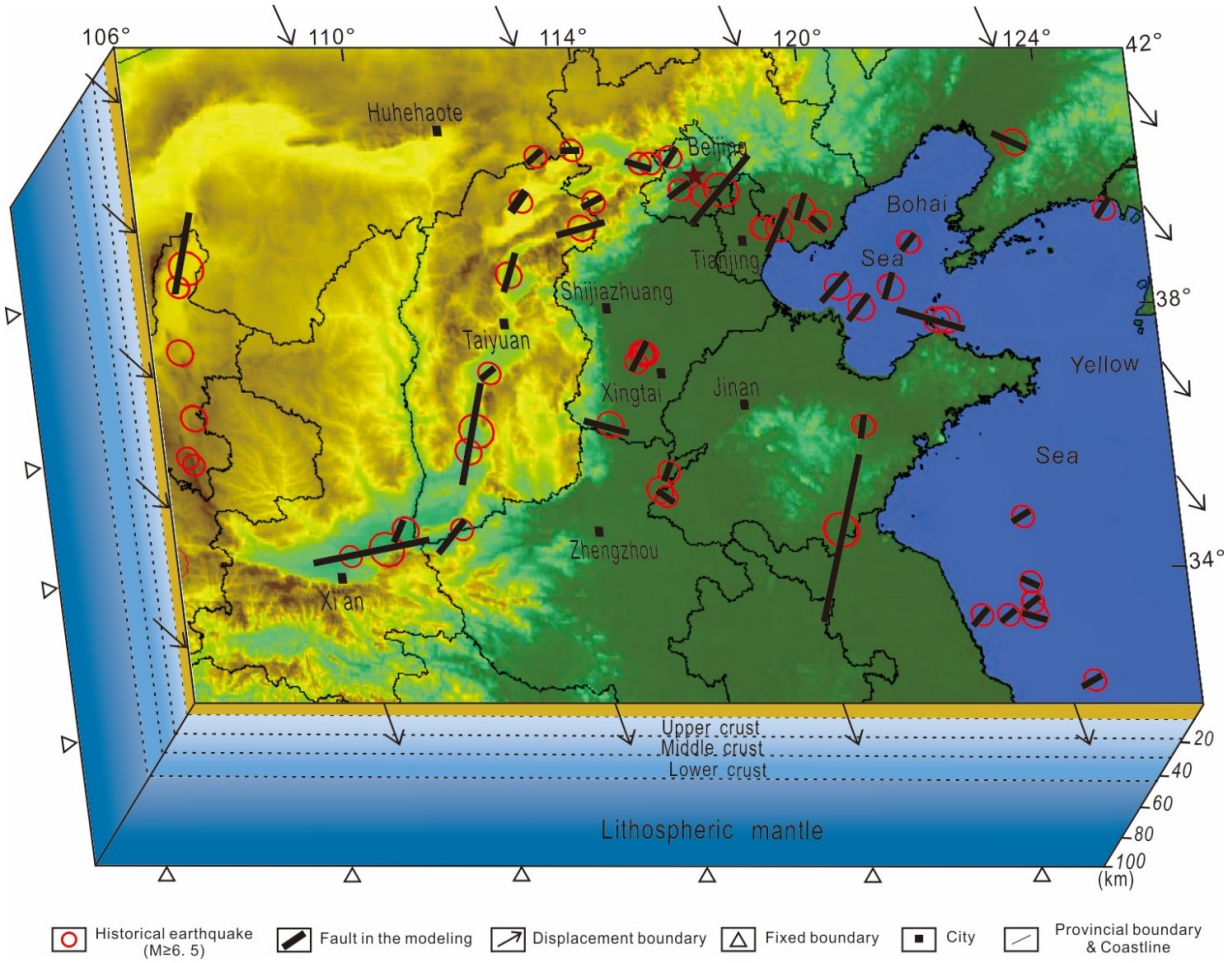
Geometry and boundary conditions of the model. The black lines depict the distribution of major seismogenic faults in the model (after Shen et al., 2004). Red circles mark the locations of earthquakes (M≥6.5) since 1303; white arrows depict displacement directions of the model boundaries. The surface of the model is set free, the bottom horizontally and vertically fixed to velocity zero.

The 3D model includes the main seismogenic faults associated with devastating earthquakes (M≥6.5) in Central North China Block since A.D. 1303 [38] (Fig 4), since the accuracy of the seismic parameters before 1303 is poor.

Earthquake location, magnitude and intensity data are from the Chinese Earthquake Catalog[39, 40]. The length of the seismogenic faults is mainly controlled by the empirical intensity-fault length equation, which is derived from the statistics for historical earthquakes in North China[38] and this equation is:

L=23.2+0.488T (6)

For historical earthquakes with magnitude only and no seismic intensity distribution data, the empirical magnitude-aftershock area length equation of North China is used:

LgL=-0.421+0.301M (7)

where L is the length of the seismogenic fault, T is the length of the seismic intensity zone VIII and M is the Richter-scale magnitude. The main fault details are recorded in Table 3.

**Table 3:** Main fault details in the 3D model.

Fault number	Time of the corresponding earthquake (year.month.day)	Epicenter	Longitude (°)	Latitude (°)	Magnitude Ms	Length (km)
F1	1556.2.2	Huaxian, Shaanxi	109.7	34.5	8.25	208.6
F2	1303.9.25	Hongdong, Shanxi	111.7	36.3	8	177.9
F3	1668.7.25	Tancheng, Shandong	118.5	34.8	8.5	291.6
F4	1668.7.26	Anqiu, Shandong	119.2	36.4	6.75	40.8
F5	1597.10.6	Bohai Sea	120	38.5	7	48.5
F6	1548.9.22	Bohai Sea	120.8	38.2	7	118.8
F7	1683.11.22	Yuanping, Shanxi	112.7	38.7	7	70
F8	1305.5.11	Huairen, Shanxi	113.1	39.8	6.5	34.3
F9	1673.10.18	Tianzhen, Shanxi	113.5	40.5	6.5	34.3
F10	1628.10.7	Huaian, Hebei	114.2	40.6	6.5	34.3
F11	1679.9.2	Sanhe-Pinggu, Hebei	117	40	8	148.6
F12	1976.7.28	Tangshan, Hebei	118	39.4	7.8	80

In the 3D model, faults were set as discrete planes of weakness cutting the FE model. These planes were described by so-called contact elements, which were defined at opposite sides of preassigned faults[41, 42]. The faults were set to propagate to the bottom of the upper crust without considering the fault occurrence in the model. The coefficient of friction of the faults was taken to be 0.4 throughout our calculations.

### Boundary conditions

Boundary conditions were applied based on GPS data and the inverse method[23, 43], as follows: the surface of the model was free; the bottom of the model was horizontally and vertically fixed to velocity zero; SE-directed displacements were set on the left and right boundaries; and ESE-directed displacements were set on the north and south boundaries. The magnitude of the displacements varied according to the GPS data[44] (Fig 4).

### Modeling results

Through the 3D viscoelastic model, investigations were made into the distribution of the tectonic stress field controlled by active fault interactions in the CNCB[23]. The 3D model is to reveal how the laws from the 2d fault interaction models controlled stress evolution and earthquakes in the CNCB. Also, the results from the 3D model can be a verification of the 2d fault interaction models.

**Fig 5:**
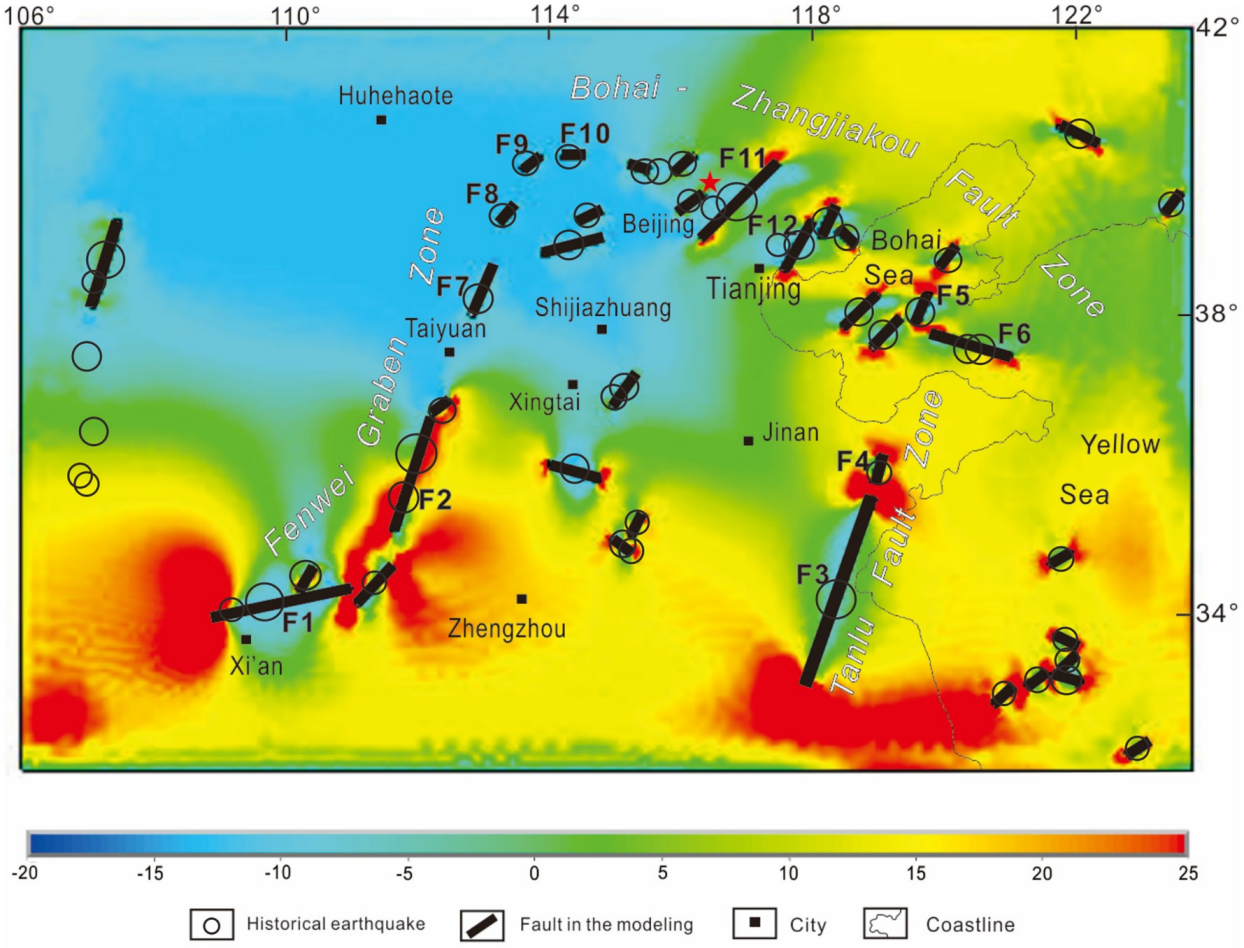
Maximum principal strain strain values calculated by the model and historical earthquakes in the CNCB. Black lines denote the seismogenic faults. An approximately N-S-trending extension is located in the southeast part of the research area, while an approximately N-S-trending shortening is located in the northwest part.

**Fig 6:**
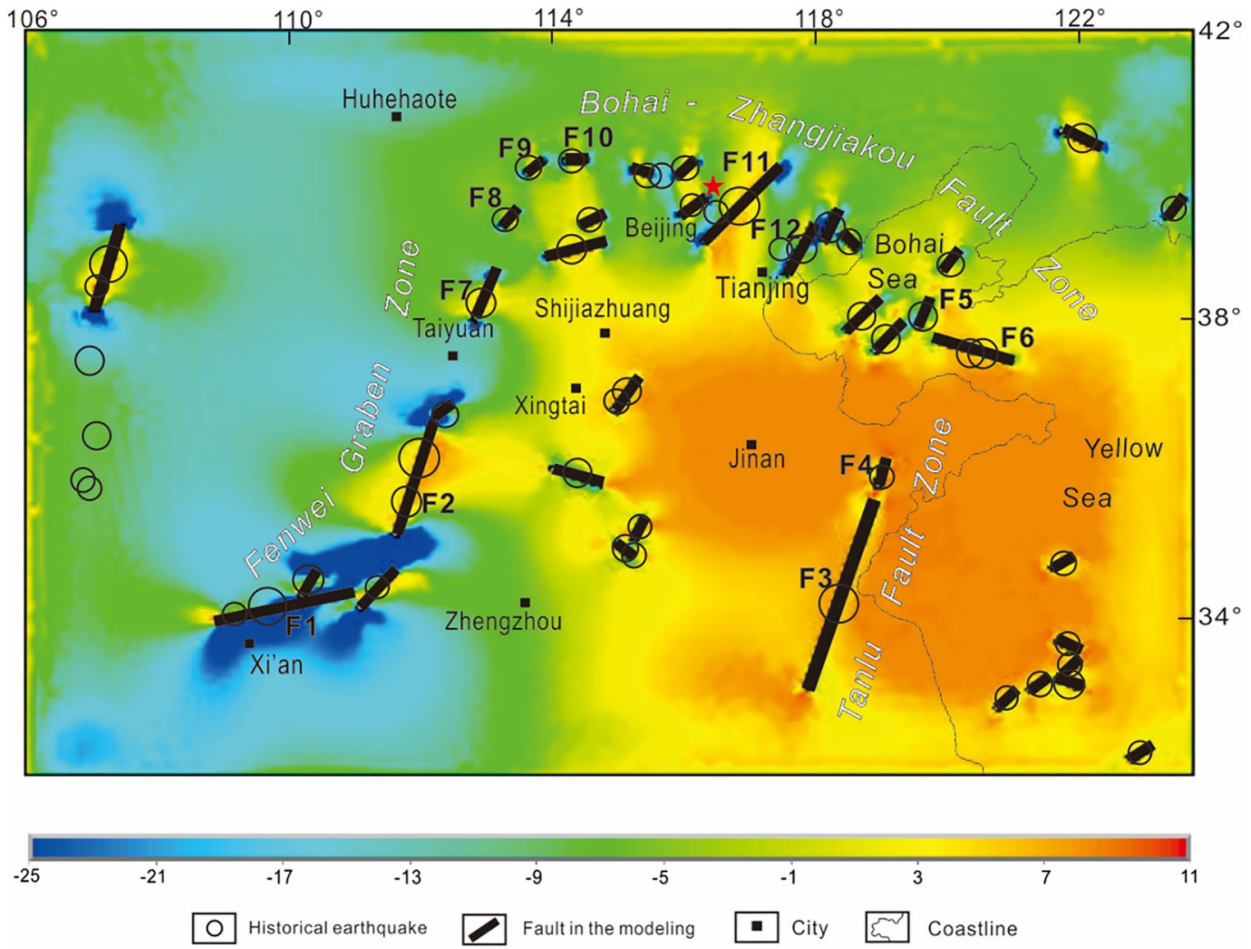
Minimum principal strain value calculated by the model and historical earthquakes in the CNCB. Black lines denote the seismogenic faults. An approximately E-W-trending extension is located in the east-central part of the research area, while an approximately N-S-trending shortening is located in the west part.

Figs 5 and 6 show the maximum and minimum principal strain calculated with the 3D model in the CNCB. On the maximum principal strain figure, an approximately N-S-trending extension is located in the southeast part of the research area, while an approximately N-S-trending shortening is located in the northwest part (Fig 5). On the minimum principal strain figure, an approximately E-W-trending extension is located in the east-central part of the research area, while an approximately N-S-trending shortening is located in the west part (Fig 6). The modeling results are consistent with the phenomenon that the North China Plate escapes to the southeast and with the topography of the eastern basin and western plateau, which is due to the Indian Plate subducting beneath the Eurasian plate and blocking from the Siberian Plate.

**Fig 7:**
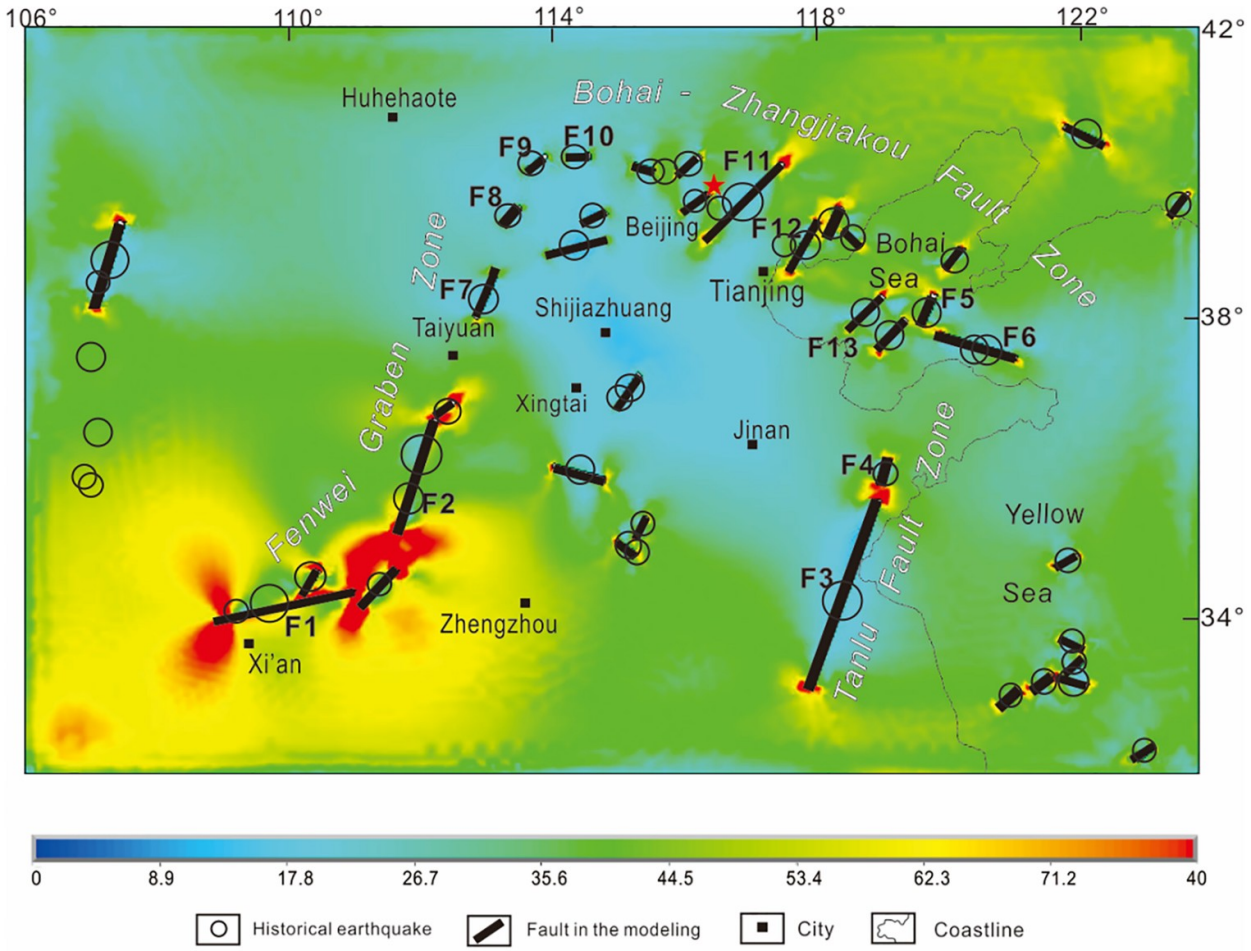
Maximum shear strain calculated by the model and historical earthquakes in the CNCB. Black lines denote the seismogenic faults. The highest strain value is located in the southern part of the Fen-wei Graben Zone. The lowest strain value is located in the northern part of the Fen-wei Graben Zone. Moderate strain values are located in the Bohai-Zhangjiakou Fault Zone and the Tan-lu Fault Zone.

**Fig 8:**
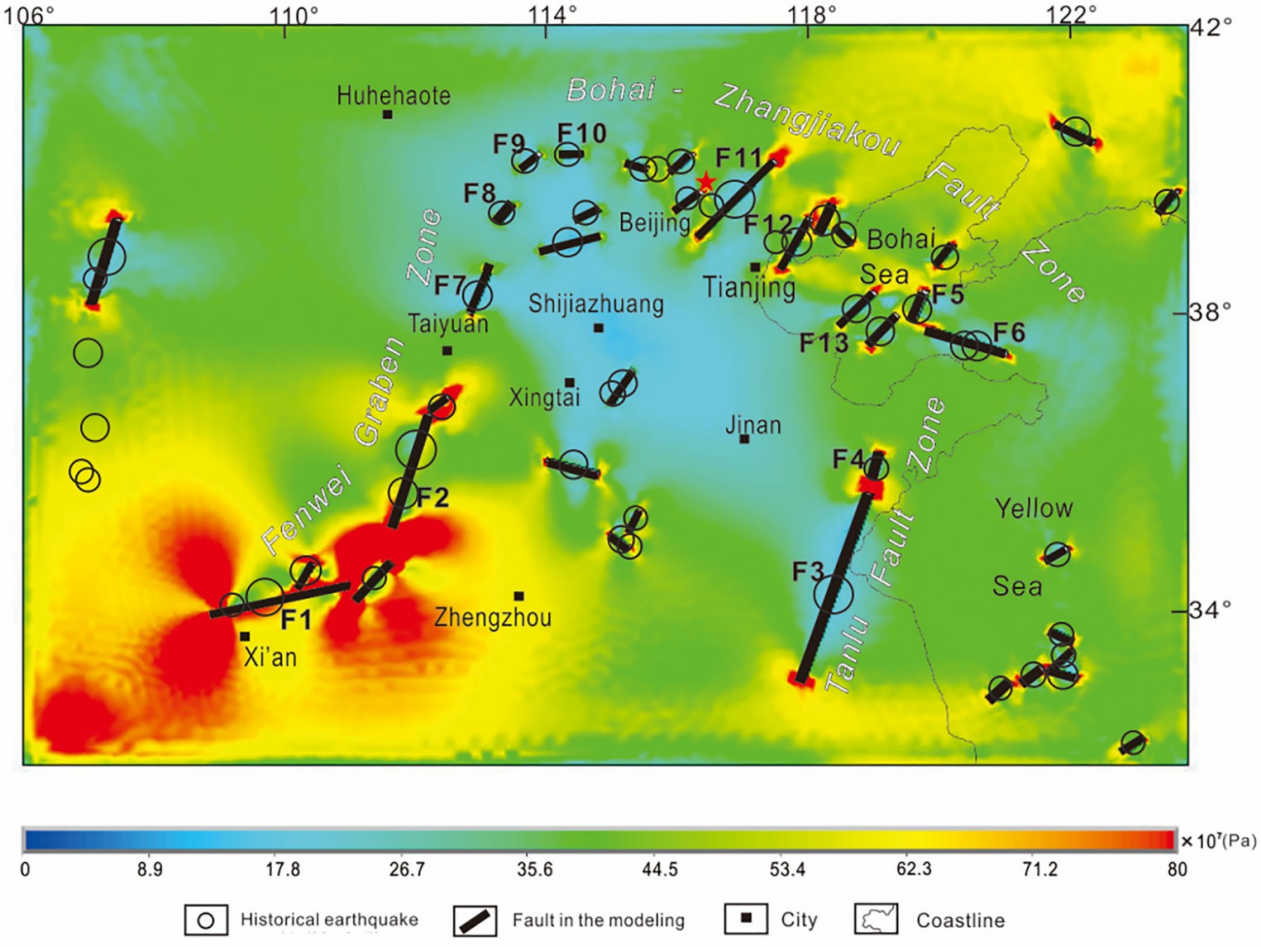
Differential intensity stress (measured in Pa) calculated by the model and historical earthquakes in the CNCB. Black lines denote the seismogenic faults. The highest stress value is located in the southern part of the Fen-wei Graben Zone. The lowest stress value is located in the northern part of the Fen-wei Graben Zone. Moderate stress values are located in the Bohai-Zhangjiakou Fault Zone and the Tan-lu Fault Zone.

The maximum shear strain and differential stress intensity triggered by stick slip and steady-state slip along the seismogenic faults are presented in Figs 7 and 8. The differential stress and maximum shear strain at a depth of 10 km are calculated because most large earthquakes occur at this depth in the CNCB. Higher differential stress and maximum shear strain could result in new active faults and more earthquakes, which indicates the range and tendency of stress changes controlled by fault interactions[17].

The laws from the model results of the conceptual model can be verified in the 3D model. For instance, the “fault length” model can be used to explain the higher differential stress and maximum shear strain values in the southern part of the Fen-wei Graben Zone south of Taiyuan. The zone with the highest differential stress and maximum shear strain values in the CNCB can be explained by the longer faults, shorter center distances, shorter overlap lengths and larger opening angles of F1 and F2 (Fig 8). The moderate value between F3 and F4 in the Tan-lu Fault Zone can be explained by the “fault length” model as well. Although F3 is the longest fault in the CNCB and the opening angle between F3 and F4 is nearly 180°, the short length of F4 is the main reason for the moderate stress value in the zone between F3 and F4. The echelon distance model can be used to explain the relatively high stress values in the Tangshan Fault Zone, in which moderate faults with short echelon distances, such as F5 and F6, are distributed. The “center distance” and the “fault length” model can be used to explain the low stress values in the northern part of the Fen-wei Graben Zone north of Taiyuan (Fig 8), in which short faults with relatively long center distances and longer echelon distances, such as F8, F9 and F10, are distributed. The “overlap length” model can be used to explain the relatively moderate stress values in the slipping zones between echelon faults in the Bohai-Zhangjiakou Fault Zone, such as the zone between F11 and F12, which are relatively long faults with a large overlap length and short normalized center distance (Fig 8).

## Discussion

Factors affecting the mechanisms of large earthquakes in the CNCB mainly include the coupling of the velocity anomaly body and active faults[45–47], the constraints on the boundaries of different active blocks[48, 49] and the anisotropy of the lithosphere[50]. The current seismic hazard assessment relies on the characteristic earthquake recurrence model, i.e., the detailed locations of the active faults[51, 52]. However, most of the aftershock and clustering earthquake sequences do not recur on the main seismogenic fault but are controlled by the fault interactions of the adjacent seismic structures [53]. Additionally, the mechanism of newly formed active faults is the result of interactions, such as the formation of the San Andreas Fault at the Mendocino triple junction [54].

Problems involving fault interactions have received much attention through various analytical methods and physical and digital simulations over the past decades. However, uncertainty exists in the way that the geometry of the seismogenic faults controls the distribution of stress fields and earthquakes. We generated four groups of conceptual models to reveal the influences of the fault length ratio, center distance, overlap coincidence ratio and opening angle in our study.

In the 3D viscoelastic model of the CNCB, seismogenic faults are used instead of shallow active faults to study fault interactions. The distribution of shallow active faults, which has been used in some models of the CNCB[55, 56], is the shallow manifestation of deep seismogenic structures. Unlike the active faults, the seismogenic faults have magnitudes that are more proportional to the intensity of the earthquake and the degree of influence on the stress field.

The factors of fault interaction should be considered in long-term seismic hazard assessment in these zones. The calculated results reveal a well-matched highest stress concentration between Xi’an and Taiyuan, which can be explained by the longer faults, shorter center distances, shorter echelon distances, shorter overlap lengths and larger opening angles of the faults in the zone. Between Beijing and Tianjin, the shorter fault lengths, longer center distances, longer overlap lengths and smaller opening angles lead to lower stress concentrations. The further work need to do is quantificationally analysis fault interaction, such as works from Liu and Konietzky [57, 58].

## Conclusions

The conceptual models considered the influences of the fault length ratio, center distance, overlap coincidence ratio and opening angle, and the following primary conclusions are drawn:

1. For two equal-length collinear faults, the interaction is larger as the interacting faults are more closely positioned. Additionally, the modeling makes it clear that the interaction is significant only when the slipping zones are closer than half of the fault length.
2. The interaction in the slipping zone increases sharply as the inner tips approach each other and decreases after the inner tips pass each other to form a larger overlap ratio. Additionally, compared with Model Group A, fault interaction is stronger as the inner tips pass each other, which impedes their growth after some degree of overlap. Moreover, fault interaction is stronger as the echelon distance becomes shorter. Only when the echelon distance is approximately one-fifth of the fault length is the calculated differential stress in the overlap area larger than that on the far ends.
3. For two unequal-length collinear faults, the interaction is stronger as one fault becomes longer. The result emerges that a large preexisting slip zone can reactivate slip and movement along a nearby smaller slip zone. In other words, segmentation of fault slip zones can substantially affect the interpretation of observations[9].
4. The fault interaction at the slipping zone becomes stronger as the opening angle approaches 180°. This result indicates that collinear faults are more likely to coalesce than faults with an opening angle. In other words, when the interval is constant, the risk of a seismic gap is greater when the faults on both sides are collinear. This factor should be taken into account in long-term seismic hazard assessment of a seismic block zone, background gap and preparatory gap.

Based on geological evidence and previous research in North China, the stress field is calculated by the finite element method for a 3D viscoelastic model to reveal the mechanism of seismic fault interactions. The interactions of seismic faults in Central North China Block can be explained by conceptual models. The calculated results reveal well-matched highest stress and maximum shear strain concentrations for Central North China Block located in the southern part of the Fen-wei Graben Zone, which can be explained by the longer faults, shorter center distances, shorter overlap lengths and larger opening angles of the faults in this area. The lowest stress concentration for Central North China Block located in the northern part of the Fen-wei Graben Zone can be explained by the short fault lengths and long center distances. In the Bohai-Zhangjiakou Fault Zone, longer center distances, longer overlap lengths and smaller opening angles lead to moderate stress concentrations. The relatively moderate stress values in the Tan-lu Fault Zone can be explained by interactions between short and long faults.

The factors of fault interactions and calculated stresses under the present-day stress field distribution should be considered in long-term seismic hazard assessment in North China. The southern part of the Fen-wei Graben Zone may have a relatively high seismic risk in the future.
